# Adherence to home fortification with micronutrient powders in Kenyan pre-school children: self-reporting and sachet counts compared to an electronic monitoring device

**DOI:** 10.1186/s12889-018-5097-2

**Published:** 2018-02-01

**Authors:** Emily M. Teshome, Veronica S. Oriaro, Pauline E. A. Andango, Andrew M. Prentice, Hans Verhoef

**Affiliations:** 1 MRCG Keneba at MRC Unit The Gambia, and MRC International Nutrition Group, London School of Hygiene and Tropical Medicine, London, UK; 2 Maseno University, School of Public Health and Community Development, Maseno, Kenya; 3 Cell Biology and Immunology Group and Division of Human Nutrition, Wageningen University, Maseno, The Netherlands; 4 Faculty of Epidemiology and Population Heath, London School of Hygiene and Tropical Medicine, Keppel Street, London, WC1E 7HT UK

**Keywords:** Child, Preschool, Patient compliance, Food, fortified, Iron, Kenya, Self-report

## Abstract

**Background:**

The efficacy of home fortification with iron-containing micronutrient powders varies between trials, perhaps in part due to population differences in adherence. We aimed to assess to what extent adherence measured by sachet count or self-reporting forms is in agreement with adherence measured by electronic device. In addition, we explored how each method of adherence assessment (electronic device, sachet count, self-reporting forms) is associated with haemoglobin concentration measured at the end of intervention; and to what extent baseline factors were associated with adherence as measured by electronic device.

**Methods:**

Three hundred thirty-eight rural Kenyan children aged 12-36 months were randomly allocated to three treatment arms (home fortification with two different iron formulations or placebo). Home fortificants were administered daily by parents or guardians over a 30 day-intervention period. We assessed adherence using an electronic device that stores and provides information of the time and day of opening of the container that was used to store the fortificants sachets in each child’s residence. In addition, we assessed adherence by self-reporting and sachet counts. We also measured haemoglobin concentration at the end of intervention.

**Results:**

Adherence, defined as having received at least 24 sachets (≥ 80%), during the 30-day intervention period was attained by only 60.6% of children as assessed by the electronic device. The corresponding values were higher when adherence was assessed by self-report (83.9%; difference: 23.3%, 95% CI: 18.8% to 27.8%) or sachet count (86.3%; difference: 25.7%, 95% CI: 21.0% to 30.4%). Among children who received iron, each 10 openings of the electronic cap of the sachet storage container were associated with an increase in haemoglobin concentration at the end of intervention by 1.2 g/L (95% CI: 0.0 to 1.9 g/L). Adherence was associated with the age of the parent but not with intervention group; with age, sex or anthropometric indices of the child; or with age or sex of the parent or guardian.

**Conclusions:**

The use of self -reporting and sachet count may lead to overestimates of adherence to home fortification.

**Trial registration:**

The trial was registered with ClinicalTrials.gov (NCT02073149) on 25 February 2014.

**Electronic supplementary material:**

The online version of this article (10.1186/s12889-018-5097-2) contains supplementary material, which is available to authorized users.

## Background

In 2011, WHO recommended home fortification with iron-containing micronutrient powders for children aged 6-23 months in areas with a prevalence of anaemia exceeding 20% [1]. The basis of this recommendation was a meta-analysis of randomised controlled trials showing moderate quality evidence for an effect on anaemia and haemoglobin concentration [2]. In a recent placebo-controlled trial, however, we failed to find effects of home fortification with iron-containing micronutrient powders on either anaemia or haemoglobin concentration [3]. In addition, in an updated meta-analysis, we showed that the magnitude of the effect on haemoglobin concentration varied between trials [3]. Such variability in trial results may be due in part to population differences in adherence to home fortification. In this paper, we conceive of adherence as the parent’s or guardian’s act of administering home fortificants to their child in accordance with our recommendations about timing, dosage, frequency and method. Adherence has been assessed in several trials by self-reporting (or proxy reporting, in the case of guardian reporting for children in their care) and sachet counts. Although these assessment methods are easy to use and relatively inexpensive, their reliability and validity are controversial [4]. Both assessment methods frequently result in overestimation of adherence because study participants respond in a manner that they believe is socially desirable but conceals their actual behaviour, or participants may consciously or unconsciously fail to return unused medication. Poor recall, particularly over long periods, can also lead to underreporting and underestimation of adherence [4–6].

A medication event-monitoring system (MEMS) is a battery-operated device in a cap that fits the bottle containing drugs or supplements. Its built-in microprocessor records and stores date and time of all openings. Although expensive, adherence assessment using MEMS is superior to medication counts and self-reporting [7, 8] and it has been considered to be the reference standard in several trials [9, 10]. We are not aware of studies that used MEMS to measure adherence to home fortification with micronutrient powders.

We aimed to assess to what extent adherence measured by sachet count or self-reporting forms is in agreement with adherence measured by electronic devices. In addition, we explored how each method of adherence assessment (electronic device, sachet count, self-reporting forms) is associated with haemoglobin concentration measured at the end of intervention; and to what extent baseline factors were associated with adherence as measured by electronic device.

## Methods

### Study design

This report concerns a secondary analysis of an explanatory, individually-randomised, placebo-controlled non-inferiority trial to assess the effect of daily home fortification on haemoglobin concentration and iron status. We measured adherence over the 30 day-intervention period using MEMS, self-reporting and sachet counts. Details regarding study methodology are available in the study protocol [3].

### Study area and participants

The study was conducted in 3 administrative areas of Kisumu West, Kenya, in the period between January and December 2014. A total of 433 children aged 12-36 months were invited by field workers to the research clinic for screening and their parents signed informed consent forms. During the screening period, medical staff verified their ages through birth certificates or health cards, conducted medical examination, took anthropometric measurements (height and weight), and collected venous blood (4 mL) in tubes containing Li-heparin after 30 days of intervention for subsequent determination of haemoglobin concentration (HemoCue 301, Ängelholm, Sweden).

Children who attained the following admission criteria were randomised: aged 12–36 months; resident in the study area and parents intending to stay in the area for the subsequent 9 months; child had no twin; parental consent form signed by both parents; not acutely sick or febrile (axillary temperature ≤ 37 °C) at the time of recruitment; absence of reported or suspected major systemic disorder; haemoglobin concentration ≥ 70 g/L; not severely wasted (weight-for-height z-score > − 3 SD); no known allergy to dihydroartemisinin-piperaquine, benzimidazole or praziquantel; no reported history of using anti-helminth drugs in the 1-month period before the screening date; not at risk of malaria (e.g. because children had received chemoprophylaxis).

To protect children against malaria and severe anaemia during the intervention period, we administered pre-medications to every eligible child at the end of the screening visit. We gave a therapeutic course of dihydroartemisinin-piperaquine (Sigma-tau, Rome, Italy; target dose: 40 mg dihydroartemisinin/320 mg piperaquine), albendazole (Indoco Remedies, Mumbai, India) and praziquantel (Cosmos, Nairobi, Kenya) [1, 11].

### Interventions and follow-up

Three days after starting pre-medication, parents were asked to bring their children again for a visit to the research clinic, where children were randomly allocated to receive daily home fortification for 30 days with micronutrient powders containing: a) 3 mg iron as NaFeEDTA, or b) 12.5 mg iron as encapsulated ferrous fumarate, or c) placebo (no iron). The micronutrient powders were manufactured specifically for this trial by DSM Nutrition Products (Johannesburg, South Africa) and packaged in 1 g sachets. The home fortificant powders for all three groups contained the same multiple micronutrients other than iron (Table 1) as recommended by the Home Fortification Technical Advisory Group, except folic acid, which we omitted because of our concerns that it may be utilized by *Plasmodium* parasites and increase failure risk of antifolate drugs [12]. We do not believe that exclusion of folic acid from the fortificants affects the generalisability of our results.

**Table 1:** Formulation of micronutrient powders

Micronutrient	Content
Vitamin A, μg RE	300
Vitamin D, μg	5
Vitamin E, mg	5
Vitamin C, mg	30
Thiamin (vitamin B_1_), mg	0.5
Riboflavin (vitamin B_2_), mg	0.5
Niacin (vitamin B_3_),mg	6
Vitamin B_6_ (pyridoxine), mg	0.5
Vitamin B_12_ (cobalamine), μg	0.9
Iron	
EITHER iron as encapsulated ferrous fumarate, mg	12.5
OR iron as NaFeEDTA, mg	3
OR no iron (placebo)	0
Zinc, mg	5
Copper, mg	0.56
Selenium, μg	17
Iodine, μg	90

The micronutrient powders were packed in 1-g plain white foil single-serve sachets that were identical in appearance and that did not result in apparent differences in taste, texture or colour of *uji* (a gruel made from locally milled flour from either maize or sorghum grains that is commonly given to young children in the study area). The MEMS device comprised of a white plastic bottle with an electronic cap and had no other marker except the serial number, label with the child’s identification number, date of start and end of intervention period. Except for the trial coordinator and one field supervisor, neither parents nor research assistants were informed about the function of the electronic device. Instead they were informed that the MEMS cap is essential for maintaining the moisture content and good hygienic conditions of the micronutrient powders.

The research assistants gave parents a supply of 30 sachets in a plastic bottle with a MEMS electronic cap and instructed them to add the contents of one sachet to the child’s semi-solid, ready-prepared foods for a period of 30 days on a daily basis. Pre-school children mostly consume *uji*. The first dose of the treatment was administered at the research clinic. Parents were shown how to mix the micronutrient powder with *uji*. Parents were trained by the research assistants to fill out the self–reporting form (Additional file 1) and were instructed to fill out the form each day after the child finished consuming a meal containing the micronutrient powders. They were shown how to pack the empty sachet in an empty zip-lock plastic bag provided to them and instructed to immediately report any sickness or adverse reactions experienced by the child during the intervention period. Each child was issued with a plate, a spoon and a mug to minimise sharing of the sachet content. A return date to the research clinic was written on the self-reporting forms, plastic bag and MEMS bottle, and parents were verbally informed about this return date.

Research assistants conducted pre-announced home visits (one visit per child per week), for a total of 3 visits per child. These visits were denoted as ‘regular monitoring’ to check if the child was still in the study area, if parents were following protocol when administering the fortificants, if sachets were still in the MEMS device (research assistants were instructed not to open the bottle during the home visits but to gently shake the bottle to ascertain the contents), and to check if parents were filling out forms and storing empty sachets. The research assistants also discussed problems or clarified procedures, but they did not give parents instructions additional to those given during the randomisation visit. All observations and problems experienced by parents were recorded in a home-visit report form and submitted to the field supervisor at the end of each day. Sick children were referred to the research clinic.

Parents were asked to bring their children back to the research clinic at the end of the 30-day intervention period, where phlebotomists collected blood and performed point-of-care tests (including haemoglobin concentration) using the same procedures as in the screening visit. Research assistants collected all the adherence-measuring tools.

### Adherence measuring tools

#### Medication events monitoring system (MEMS)

We used an electronic monitoring and time-recording device (MEMS 6 TrackCap 45 mm without LCD display; http://www.medamigo.com/products/mems-cap) that was given for the duration of the study to parents of participating children. This battery-operated device stores and provides detailed information of the timing of the events, e.g. day and time intervals between openings, and percent of prescribed doses taken [13]. Each bottle had a capacity of holding 30 micronutrient sachets, and was labelled with a child’s identification number, serial number of the electronic cap, name of the child, and a start and end date for ease of identification and tracking. Parents were thoroughly instructed to open the bottle only when removing the sachet and to close it immediately after each opening. The research assistant demonstrated this instruction when the first dose of powder was administered at the research clinic. The information recorded in the MEMS cap was downloaded at the end of the 30-day intervention period, and stored electronically in the computer.

#### Sachet counts

We defined sachet count as the number of empty sachets stored at the end of the intervention period. The empty sachet was considered to be a proxy indicator that child has consumed the content of a full sachet. Research assistants instructed parents to securely keep empty sachets in a zip-lock plastic bag marked with the child’s name and identification number. On their return visit to the research clinic, parents returned the empty sachets contained in the zip-lock plastic bag, plus any left-over full sachets in the MEMS device. The research assistant immediately counted and recorded the empty sachets into the Excel spread sheet. The leftover full sachets remained in the MEMS bottle and were only removed after the MEMS data had electronically been transferred to PowerView software (AARDEX Group Ltd., Sino, Switzerland). The number of full sachets was manually recorded in the MEMS PowerView data sheet to verify if the number of times the MEMS cap was opened matched the sachet count.

#### Self-reporting form

We used a 1-page self-reporting form (Additional file 1) that was simple and easy to fill out by a parent, even with low level of primary education. The form had a simple chart that allowed a parent to easily tick a box whenever a full sachet of micronutrient powder was administered to the child, either in the morning, mid-morning, lunch, mid-afternoon or evening. The form was translated into the local language (*dholuo*).

### Definition of endpoints

Adherence to treatment was defined as the number of days within the intervention period that the electronic monitoring device was opened, the number of empty sachets within the intervention period, or the number of days within the intervention period that the child received home fortification as reported by the mother or guardian (designated by number of ticks on the self-reporting form). High adherence was defined as adherence ≥ 80% (24 days or more). This threshold is arbitrary but is often used in published studies on medication adherence [8, 13–16]. Low-adherence was defined as adherence < 80% (23 days or less).

### Statistical analysis

Analysis was conducted using SPSS 21 (IBM, Armonk, NY), WHO Anthro software vs.3.2.2 (World Health Organisation, Geneva, Switzerland), PowerView software vs3.5.2, R-software version 3.2.0 (https://www.r-project.org) and CIA software (https://www.som.soton.ac.uk/research/sites/cia/download/).

For children who did not complete the intervention because parents refused supplementation after randomisation, or due to unknown reasons, we retained 30 days of intervention, or 30 sachets, as denominators of these proportions. For children who were lost to follow-up during the intervention period because they had moved out of the study area, we initially intended to censor the observation period at the day that the child was lost and to use proportional weights to account for differences in observation time between children. However, because we could not establish the day that these children moved, we excluded them from the analysis.

Differences in proportions of children with high adherence were compared by Newcomb’s method for paired samples. To evaluate agreement in adherence, measured as continuous variables between various assessment methods, we used scatter plots with corresponding Pearson’s product-moment correlation coefficient, r. We used t-tests and linear regression analysis to model haemoglobin concentration at the end of 30 days of intervention as a function of adherence. In these analyses, we excluded children who received placebo.

Lastly, we used simple beta regression analysis with a logit link to identify variables that were associated with adherence as measured by the MEMS device. In this analysis, we transformed the adherence outcome variable so that fractional response values of 0 or 1 were replaced using the formula *y*^′^ = [*y* + (*n* − 1) + 0.5]/*n* [17] with *y* being the fractional response, and *n* being the sample size. The variables examined included experimental treatment, baseline characteristics of the child (sex, age, being infected by *P. falciparum*, wasting, stunting), the child being sick during the 30-day intervention period, and characteristics of the parent or guardian (age, gender, education level).

## Results

### Participant flow and baseline characteristics

**Fig. 1:**
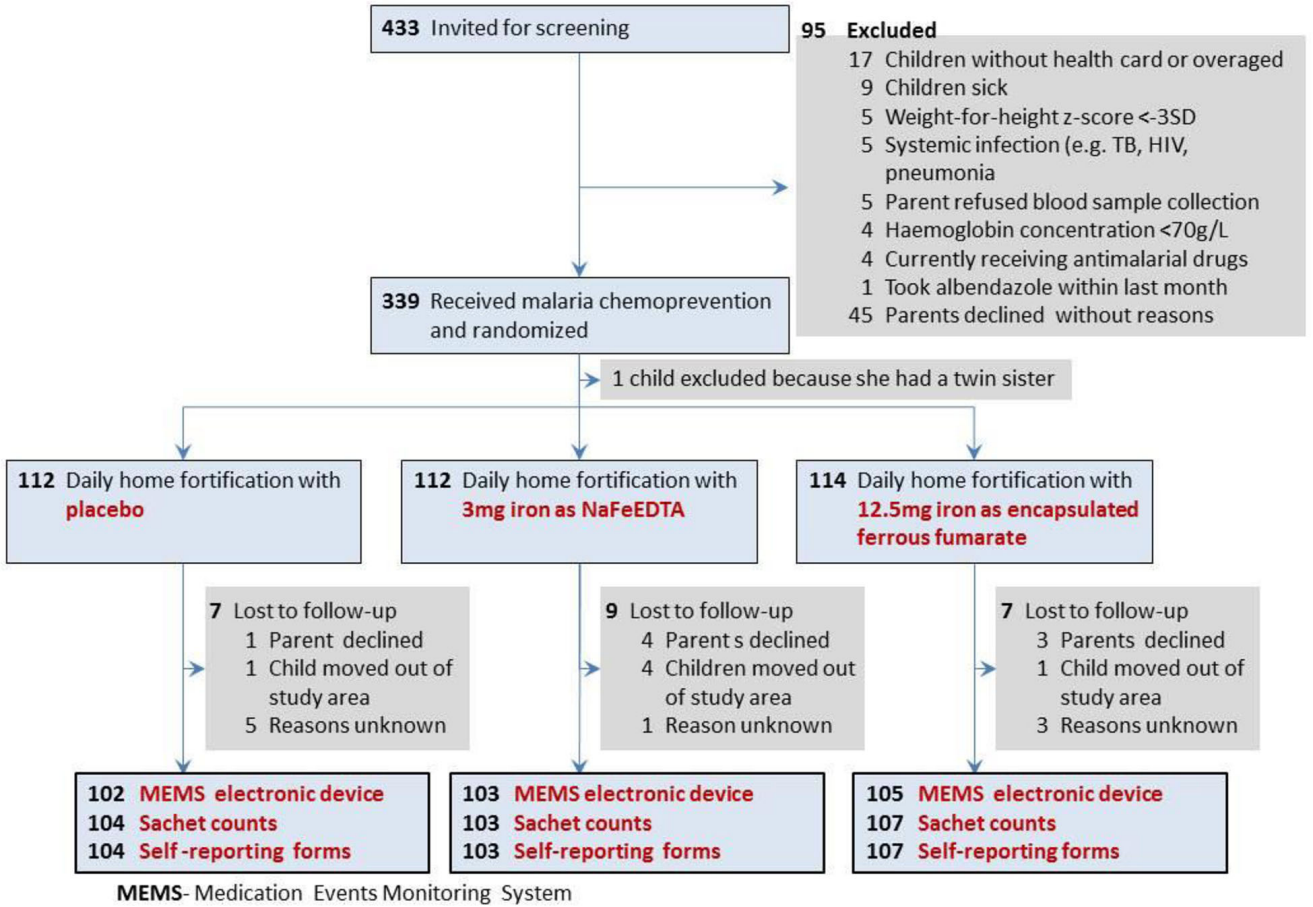
Participant flow chart

The profile of trial participants and reasons for loss to follow-up are presented in Fig. 1. Of 433 children invited for screening at the research clinic, 339 were eligible. One child was excluded after randomisation because she was found to have a twin sister. Twenty-three children were lost to follow-up: parents or guardians from eight children refused further cooperation, six children moved residence, and nine children were lost for unknown reasons. Fifteen participants failed to submit their self-reporting forms, 13 participants did not return empty sachets, one electronic cap was damaged and another was lost. All parents who declined to continue with the study submitted their measuring instruments and gave permission for data collected to be included in the study.

Table 2 presents baseline characteristics of study participants. We included more boys than girls (186 versus 152), mean age (SD) was 23.6 ± 6.5 months and the distribution of children by age class was evenly distributed between the intervention groups. At baseline, the mean (SD) haemoglobin concentration was 105 (3.2) g/L and 62.1% (209/338) of the children were anaemic. The prevalence of being stunted, wasted and underweight were 30.2% (102/338), 2.9% (10/338) and 13.6% (46/338), respectively. Mean (SD) age of primary guardians was 27.4 (6.3) years ranging from 16 to 50 years. Interviews conducted at end of study (post intervention) showed that about 80.2% (158/197) of the mothers administered the sachets at home; in other cases, the child’s older siblings (9.1%), extended family members (6.1%) or father (4.1%) took up this role. Most primary guardians 71.2% (141/197) attained upper primary education level followed by secondary education level 20.8% (41/197), lower primary education level 5.5% (11/197) and very few 2.5% (5/197) attained tertiary level of education. At the end of 30 days of intervention, the mean (SD) haemoglobin concentration was 108.5 (12.7) g/L and the prevalence of anaemia was about 46%.

**Table 2:** Baseline characteristics of the study population

Characteristic	Placebo	Iron, 3 mg as NaFeEDTA	Iron, 12.5 mg as ferrous fumarate
n	112	112	114
General characteristics			
Sex, male	69 (61.6%)	61 (54.5%)	56 (49.1%)
Age, months	22.8 (6.8)	23.2 (6.2)	24.9 (6.4)
Age class			
12–23 months	61 (54.5%)	57 (50.9%)	44 (38.6%)
24–36 months	51 (45.5%)	55 (49.1%)	70 (61.4%)
Nutritional markers			
Haemoglobin concentration, g/L	104.4 (13.2)	105.9 (13.3)	104.7 (13.3)
Anaemia			
Moderate (haemoglobin concentration 70–99.99 g/L)	33 (29.5%)	34 (30.4%)	36 (31.6%)
Mild (haemoglobin concentration 100–109.99 g/L)	39 (34.8%)	31 (27.7%)	37 (32.5%)
No anaemia (haemoglobin concentration ≥ 110 g/L)	40 (35.7%)	47 (42.0%)	41 (36.0%)
Anthropometric markers			
Body height, cm	80.9 (5.9)	82.1 (5.7)	82.4 (5.1)
Body weight, kg	10.6 (1.8)	10.8 (2.0)	10.9 (1.7)
Stunted (height-for-age z-score < −2 SD)	34 (30.4%)	31 (27.7%)	37 (32.5%)
Wasted (weight-for-height z-score < − 2 SD)	3 (2.7%)	5 (4.5%)	2 (1.8%)
Underweight (weight-for-age z-score < − 2 SD)	19 (17.0%)	12 (10.7%)	15 (13.2%)

Values indicate n (%), mean (SD)

### Adherence as measured by self-reporting, sachet counts and MEMS device

**Fig. 2:**
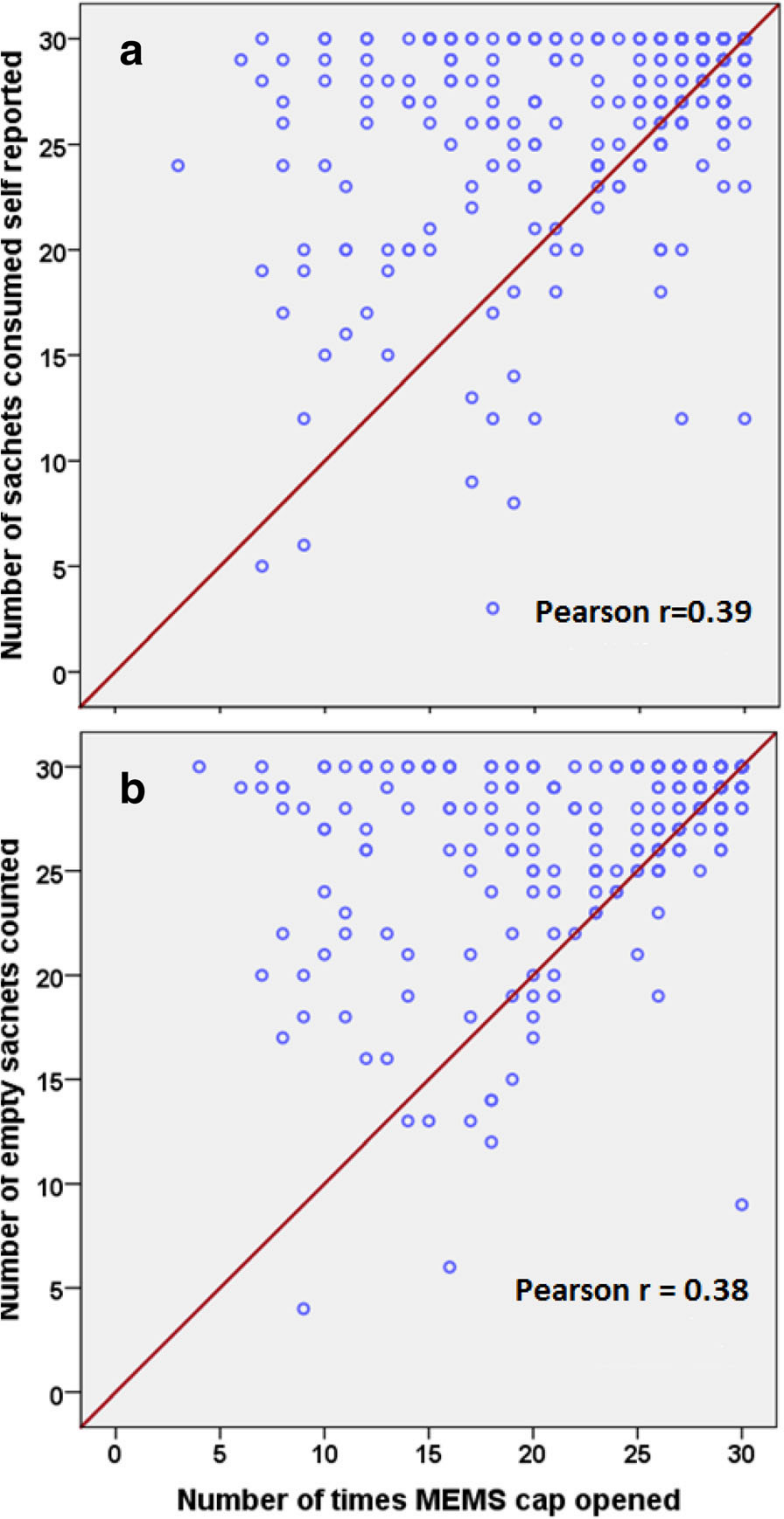
Adherence measurements as assed by self-reporting and sachet tools compared with MEMS device. Panel **a** Data were not available for 17 children because 15 self-reporting forms were either missing or not filled out and records of 2 MEMS devices missing because 1 device was damaged and another was not returned at end of 30 days after randomization. Panel **b** Data were not available for 15 children because 13 children lost or did not return empty sachets and 2 MEMS devices were unavailable. Not all data points in Panel **a** and Panel **b** are visible due to over-lapping of adherence counts. The red line indicates perfect agreement in adherence counts between pairs of assessment methods. For each panel, data points presented above the reference line indicate overestimation of adherence assessed by self-report or sachet count, respectively, as compared to adherence measured by MEMS, whereas data points presented below the reference line indicate underestimation of adherence measured by MEMS

MEMS data indicated that 60.6% of children received at least 24 sachets during the 30-day intervention period (Table 3). That level of adherence was more frequently indicated by both self-report (83.9%; difference: 23.3%, 95% CI: 18.8% to 27.8%) and sachet count (86.3%; difference: 25.7%, 95% CI: 21.0% to 30.4%) than recorded by MEMS (Table 3). The over-reporting by self-report and sachet count was apparent when inspecting the scatterplots of results obtained by these methods with those obtained by MEMS data (Fig. 2). The value for Pearson’s r expressing the strength of the correlation between adherence by self-report and MEMS device was 0.39 (*p* < 0.001), whilst the corresponding value for the correlation between adherence by sachet count and MEMS device was 0.38 (*p* < 0.001).

**Table 3:** Adherence as indicated by various assessment methods during the 30-day intervention period

Adherence	MEMS (*n* = 338)	Self-reporting (*n* = 338)	Sachet count (*n* = 338)
Coverage^a^	330 (97.6%)	317 (93.8%)	313 (92.6%)
Missing^b^	8 (2.4%)	21 (6.2%)	19 (5.6%)
High (≥ 80%, or ≥24 sachets consumed)	200 (60.6%)	266 (83.9%)	270 (86.3%)
Low (< 80%, or 1-23 sachets consumed)	130 (39.4%)	51 (16.1%)	43 (13.7%)

Values indicate n (%), *MEMS* Medication Events Monitoring System

^a^Number of children who consumed at least one of the 30 sachets scheduled during the intervention period

^b^Reasons, by intervention group: MEMS: 6 participants moved residence, 1 participant damaged the electronic device and another lost the electronic device; Self-reporting: 6 participants moved residence; 15 others failed to submit their self-reporting forms; Sachet counts: 6 participants moved residence; 13 others failed to return empty sachets

### Adherence and haemoglobin concentration at the end of iron intervention

Among 228 children who received home fortification with iron, formulated either as ferrous fumarate or NaFeEDTA, the mean (SD) haemoglobin concentration at the end of intervention was 109.3 (12.2 g/L) g/L, with a corresponding prevalence of anaemia of 44.3% (*n* = 101). Among these children, we could identify an association between haemoglobin concentration at the end of the 30-day intervention period and adherence as assessed by MEMS (*p* = 0.04), but not when assessed by self-report or sachet count (*p* = 0.11 and *p* = 0.14, respectively) (Table 4). Each 10 openings of the electronic cap were associated with an increase in haemoglobin concentration by 1.2 g/L (95% CI: 0.0 to 1.9).

**Table 4:** Association between adherence and haemoglobin concentration in children who received daily home fortification with iron as ferrous fumarate or NaFeEDTA

Adherence assessment method	Difference in haemoglobin concentration, g/L (95% CI)^a^	*P*-value
MEMS	1.2 (0.0 to 1.9)	0.04
Self-reporting	2.3 (−0.5 to 5.2)	0.11
Sachet count	2.7 (−0.9 to 6.3)	0.14

*MEMS* Medication Events Monitoring Systems

^a^Association expressed as the mean difference in haemoglobin concentration for each increment in adherence by 10 MEMS openings, or 10 sachets consumed as indicated by self-report or sachet count

When adherence was assessed by MEMS, we found that children with high adherence to home fortification with iron had higher haemoglobin concentration at the end of the 30-day intervention period than their peers with low adherence (111.1 g/L versus 106.6 g/L; difference: 4.6 g/L, 95% CI: 1.2 g/L to 8.0 g/L). There was no evidence for such an association when adherence was assessed by self-report (109.7 g/L versus 107.0 g/L; difference: 2.7 g/L, 95% CI: -1.6 g/L to 7.0 g/L) or sachet counts (109.6 g/L versus 107.3 g/L; difference: 2.2 g/L, 95% CI: -2.6 g/L to 7.1 g/L).

### Factors associated with adherence measures using MEMS device

**Fig. 3:**
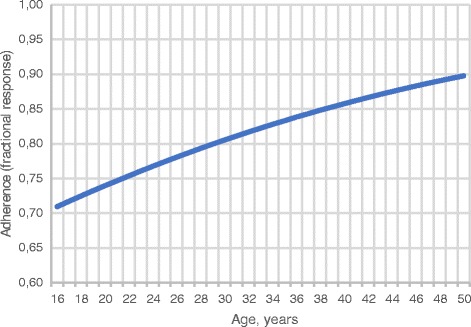
Age of parent or guardian at baseline as a predictor of adherence to daily home fortification

Adherence to home fortification was associated with the age of the parent or guardian: for each increment in age by 1 year, the logit of the fractional response increased by 4% (95% CI: 1% to 6%, *p* = 0.002; Fig. 3). Compared to adherence with home fortification with placebo, there was no evident difference in adherence with home fortification with iron, either as NaFeEDTA or as ferrous fumarate (Table 5). We also found no evidence that adherence was affected by baseline characteristics of the child (sex, age, being infected by *P. falciparum*, wasting, stunting), the child being sick during the 30-day intervention period, or characteristics of the parent or guardian (gender, education level) (Table 5).

**Table 5:** Variables associated with adherence, assessed by MEMS device

Variable	Adherence	Logit ratio (95% CI)	*p*-value
Intervention group			
Placebo	77.7%	Reference	
NaFeEDTA	79.6%	1.12 (0.84-1.49)	0.45
Ferrous fumarate	79.0%	1.08 (0.81-1.44)	0.61
Child’s sex			
Male	77.8%	Reference	
Female	79.9%	1.13 (0.89-1.43)	0.32
Child’s age, years^a^	–	0.99 (0.80-1.23)	0.92
*P. falciparum*, baseline			
Not infected	79.9%	Reference	
Infected	77.2%	0.86 (0.67-1.09)	0.21
Anthropometric indices, baseline			
Wasting (weight-for-height z-score change, 1 SD)^a^	–	1.07 (0.95-1.20)	0.27
Stunting (height-for-age z-score change, 1 SD)^a^	–	1.06 (0.97-1.15)	0.18
Underweight (weight-for-age z-score change, 1 SD)^a^	–	1.08 (0.98-1.20)	0.12
Child was sick during intervention period			
No	78.8%	Reference	
Yes	78.9%	1.01 (0.77-1.33)	0.95
Parental education			
None	85.4%	Reference	
Primary	77.2%	0.58 (0.30-1.13)	0.11
Secondary	81.8%	0.77 (0.38-1.58)	0.47
Tertiary	82.7%	0.82 (0.24-2.83)	0.76
Parent’s sex			
Male	64.1%	Reference	
Female	79.0%	2.11 (0.61-7.29)	0.24

Logit ratios indicate the change in logit relative to the reference class, or the relative change in logit associated with 1 unit increment in the^a^ continuous variables

## Discussion

Compared to adherence monitoring by electronic device, we found that self-reporting and sachet count over a 30-day intervention period was associated with over-reporting. Among children who received iron, haemoglobin concentration at the end of the 30-day intervention period was associated with adherence as assessed by MEMS, but there was no evidence of it being associated with adherence as assessed by self-report or sachet count. Each 10 openings of the electronic device were associated with an increase in haemoglobin concentration of 1.2 g/L (95% CI: 0.0 to 1.9). Adherence to home fortification was associated with the age of the parent or guardian.

We assume that assessment of adherence by electronic device is more reliable than by self-report or sachet count. This has been reported by others [7, 8] but also seemed to be confirmed in the present study by our finding that adherence assessed by MEMS was associated with a haemoglobin concentration response in children who received iron, whereas such a response could not be shown when adherence was assessed by self-report or sachet count. However, we cannot exclude the possibility that there were parents who administered missed doses on the next day in addition to the scheduled dose. Compared to ingestion of a single dose of non-haem iron, multiple dosing leads to a decrease in the proportion of iron absorbed, but an increase in the absolute amount of iron absorbed [18]. Thus, the efficacy of missing a dose and doubling it on the next day is probably in between the efficacy of regular daily dosing and skipping a missed dose altogether. In that case, assessment of adherence by sachet count may theoretically be better than by electronic device On the other hand, self-reporting and sachet counts have the limitation of being prone to social desirability bias: parents may have a tendency to overreport ‘good behaviour’, to remove but not administer sachet contents, or to underreport adherence due to inaccurate recall or loss of sachets. Such bias has been shown in many studies for self-reporting of medication (e.g., [19–21]). Thus MEMS may more actually indicate frequency of intake of home fortificants powders and thus intervention efficacy than sachet count, which is subject to social desirability bias and may be more vulnerable product use in unintended ways.

As reported elsewhere, we failed to find an effect of home fortification with either 3 mg iron as NaFeEDTA or 12.5 mg iron as ferrous fumarate on haemoglobin concentration, erythrocyte zinc protoporphyrin or plasma iron markers [3]. This failure may have been due in part to the relatively short intervention period, consumption of diets containing high phytate and phenolic compounds and in part to infection-associated inflammation, which was highly prevalent in our study population. In the present study, we found that adherence, as assessed by MEMS, showed that only 60.6% of children consumed at least 80% (≥ 24) sachets during the 30-day intervention period. These data, coupled with our finding that adherence was associated with haemoglobin concentration at the end of the 30-day intervention period indicate that sub-optimal adherence may also have contributed to our failure to show efficacy.

The association between age of the parent or guardian and adherence to home fortification should be interpreted with caution, because this finding was the result of exploratory analyses. Nonetheless, this finding supports results of a trial conducted among HIV-infected adults in Los-Angeles, where older age was associated with higher adherence rates of anti-retroviral adherence [22] compared to the younger patients. Thus, it is possible that in our present study the older parents more strictly followed instructions, resulting in improved adherence, compared to the younger carers. Further trials are needed that are specifically designed to identify and understand determinants of adherence to home fortification of micronutrient powders. In such trials, latent class analysis [23] may be helpful to define subgroups on the basis of beliefs and attitudes about home fortification at baseline.

A strong point of our study is the use of beta regression analysis to model adherence expressed as a fractional response. Linear regression analysis of the untransformed fractional response is commonly used but has limitations: a) the effect of explanatory variables tend to be non-linear, particularly with responses towards the extremes of the range [0, 1]; b) a proportion is not normally distributed (even though a normal approximation may be reasonable if all observations are reasonably close to 0.50); and c) the variance is not constant but varies with the outcome, meaning it is maximised at a population proportion of 0.50 and it shrinks when the population proportion approaches one of the boundaries. For these reasons, linear regression would be inappropriate and was not used in our analyses. Similar problems are likely to arise when estimating adherence as the mean fractional response, or when comparing groups by the mean difference in fractional response using ANOVA or assuming t-distributions. By contrast, fractional response data commonly follow a beta distribution, which can take a wide variety of shapes, depending on the parameters describing the distribution.

## Conclusions

The use of self -reporting and sachet count may lead to overestimates of adherence to home fortification. Adherence to home fortification as assessed by MEMS device was associated with the age of the parent or guardian.

## Additional file

Additional file 1: Self-reporting form. (DOCX 68 kb)

## Abbreviations

MEMS: Medication Events Monitoring System
MRC-ING: International Nutrition Group of the Medical Research Council
NaFeEDTA: Sodium Iron Ethylene-Diamine-Tetraacetic Acid
WHO: World Health Organization
