# Supplementary material for: Adherence to home fortification with micronutrient powders in Kenyan pre-school children: self-reporting and sachet counts compared to an electronic monitoring device
Source: BMC Public Health. 2018 Feb 1;18:205. doi: 10.1186/s12889-018-5097-2 (PMC5796300; doi:10.1186/s12889-018-5097-2)
Supplement: Supplementary file 1 [file 12889_2018_5097_MOESM1_ESM.docx]

SEICK PROJECT 2014

Self- Reporting Sheet

To be filled by caregiver immediately after adminstering contents in the sachets

Date of start	
ID No of Child	
Location	

End of reporting date

Tick the time child consumes warm *Uji* o r any warm meal with Micronutrient powder

Day	Morning	Mid-Morning	Lunch	Mid-afternoo	Night	Did not consume
1						
2						
3						
4						
5						
6						
7						
8						
9						
10						
11						
12						
13						
14						
15						
16						
17						
18						
19						
20						
21						
22						
23						
24						
25						
26						
27						
28						
29						
30						
